# Significant Overexpression of *DVL1* in Taiwanese Colorectal Cancer Patients with Liver Metastasis

**DOI:** 10.3390/ijms141020492

**Published:** 2013-10-14

**Authors:** Ming-Yii Huang, Li-Chen Yen, Hsueh-Chiao Liu, Po-Ping Liu, Fu-Yen Chung, Tsu-Nai Wang, Jaw-Yuan Wang, Shiu-Ru Lin

**Affiliations:** 1 Department of Radiation Oncology, Cancer Center, Kaohsiung Medical University Hospital, Kaohsiung 807, Taiwan; E-Mail: miyihu@gmail.com; 2 Department of Radiation Oncology, Faculty of Medicine, College of Medicine, Kaohsiung Medical University, Kaohsiung 807, Taiwan; 3 Cancer Center, Kaohsiung Medical University Hospital, Kaohsiung Medical University, Kaohsiung 807, Taiwan; 4 Division of Medical Research, Fooyin University Hospital, Pingtung 928, Taiwan; E-Mails: lcyen.fy@gmail.com (L.-C.Y.); fuyenchung@gmail.com (F.-Y.C.); 5 Personalized Medical Service Center, Division of Laboratory Medicine, Fooyin University Hospital, Pingtung 928, Taiwan; E-Mail: 9341@fy.org.tw; 6 Department of Surgery, Fooyin University Hospital, Pingtung 928, Taiwan; E-Mail: huang190814@gmail.com; 7 Department of Public Health, College of Health Science, Kaohsiung Medical University, Kaohsiung 807, Taiwan; E-Mail: wangtn@kmu.edu.tw; 8 Division of Gastrointestinal and General Surgery, Department of Surgery, Kaohsiung Medical University Hospital, Kaohsiung 807, Taiwan; 9 Graduate Institute of Clinical Medicine, College of Medicine, Kaohsiung Medical University, Kaohsiung 807, Taiwan; 10 Department of Surgery, Faculty of Medicine, College of Medicine, Kaohsiung Medical University, Kaohsiung 807, Taiwan; 11 School of Medical and Health Sciences, Fooyin University, Kaohsiung 831, Taiwan

**Keywords:** colorectal cancer, liver metastasis, *DVL1* overexpression, weighted enzymatic chip array (WEnCA), immunohistochemistry (IHC)

## Abstract

Undetected micrometastasis plays a key role in the metastasis of cancer in colorectal cancer (CRC) patients. The aim of this study is to identify a biomarker of CRC patients with liver metastasis through the detection of circulating tumor cells (CTCs). Microarray and bioinformatics analysis of 10 CRC cancer tissue specimens compared with normal adjacent tissues revealed that 31 genes were up-regulated (gene expression ratio of cancer tissue to paired normal tissue > 2) in the cancer patients. We used a weighted enzymatic chip array (WEnCA) including 31 prognosis-related genes to investigate CTCs in 214 postoperative stage I–III CRC patients and to analyze the correlation between gene expression and clinico-pathological parameters. We employed the immunohistochemistry (IHC) method with polyclonal mouse antibody against DVL1 to detect DVL1 expression in 60 CRC patients. CRC liver metastasis occurred in 19.16% (41/214) of the patients. Using univariate analysis and multivariate proportional hazards regression analysis, we found that *DVL1* mRNA overexpression had a significant, independent predictive value for liver metastasis in CRC patients (OR: 5.764; 95% CI: 2.588–12.837; *p* < 0.0001 on univariate analysis; OR: 3.768; 95% CI: 1.469–9.665; *p* = 0.006 on multivariate analysis). IHC staining of the immunoreactivity of DVL1 showed that DVL1 was localized in the cytoplasm of CRC cells. High expression of DVL1 was observed in 55% (33/60) of CRC tumor specimens and was associated significantly with tumor depth, perineural invasion and liver metastasis status (all *p* < 0.05). Our experimental results demonstrated that *DVL1* is significantly overexpressed in CRC patients with liver metastasis, leading us to conclude that *DVL1* could be a potential prognostic and predictive marker for CRC patients.

## 1. Introduction

Colorectal cancer (CRC) is the third most commonly diagnosed cancer in males and the second most diagnosed in females worldwide, with over 1.2 million new cases each year and 608,700 estimated deaths [1]. The clinical stage at diagnosis, site of lesion (rectum *vs.* colon), invasion of contiguous organs and presence of perforation are positive predictive factors for postoperative CRC recurrence [2]. Although there have been significant improvements in the treatment of advanced CRC as a result of using a multidisciplinary approach, individuals with postoperative recurrence or metastatic disease still have poor prognosis [2]. As many as 40%–50% of patients who undergo curative resection subsequently develop metastatic disease and die within five years [3,4]. It is suggested that undetected micrometastasis does exist, and the presence of disseminated tumor cells shed from the primary carcinoma into the circulation, before, during, or after surgery, may play a key role in relapse [5,6]. Although metastasis is the main cause of death from such tumors, the mechanism of the metastatic process in CRC is very complex and still not completely understood [7]. Hence, novel and well-characterized biomarkers would be helpful for clinicians to predict metastatic progression and prognosis of CRC patients for facilitation of therapeutic intervention.

Circulating tumor cells (CTCs) were first discovered in the blood of a cancer patient (post-mortem) by Ashworth [8]. More recently, with refined techniques and advances in molecular biology, the identification of CTCs via nucleic acid-based methodologies and PCR has developed into a useful tool in the detection of occult metastases [9]. Our recent investigations have demonstrated that the persistent presence of postoperative CTCs is a poor prognostic factor for patients with CRC after curative resection by membrane array-based multimarker assay [10–12]. In fact, we have demonstrated a high correlation between real-time quantitative-PCR and the membrane array method in the detection of CTCs in CRC patients [10]. However, the cost of the digoxigenin enzyme used for the colorimetric biochip platform was too high for routine laboratory diagnosis, and the complexity of the operation techniques have prevented its widespread utility for clinical applications. Therefore, we developed the next generation biochip operation platform—the weighted enzymatic chip array (WEnCA) platform which has now replaced the conventional digoxigenin system with the bioton-avidin enzyme system. This plays a key role in significantly lowering the overall cost [13].

The Wnt pathway (known as the wingless pathway in Drosophila) plays a role in organ development in several species, but when aberrantly activated is associated with carcinogenesis (including metastasis) [14]. Over 90% of colorectal cancers have a mutation that activates this pathway [15]. Wnt ligands bind with their target membrane receptors, frizzled and low-density lipoprotein receptor related proteins, and interfere with the multi-protein APC/β-catenin destruction complex, resulting in the downstream activation of gene transcription by β-catenin. While the complex role of β-catenin in cell proliferation and cell adhesion has been the main focus of many mechanistic studies, it is becoming increasingly evident that upstream components such as *DVL1* (Disheveled homolog 1) genes are also involved in human cancers [16,17]. However, the expression pattern of *DVL1* and its clinical significance in human colorectal adenocarcinoma have yet to be investigated.

In the first step of study, we utilized the high efficiency gene screening technology of microarrays to analyze the gene expression of 10 CRC cancer tissue specimens paired with normal adjacent tissues. From these tests we sought 31 genes that were up-regulated (gene expression ratio of cancer tissue to paired normal tissue > 2) that may serve as candidate markers. In the second step of investigation, we utilized our well-established WEnCA platform to analyze 31 up-regulated candidate genes closely related to CRC carcinogenesis in the CTCs of 214 stage I to III postoperative CRC patients’ peripheral bleed specimens. Finally, we identified immunoreactivity of DVL1 in the cytoplasm of tumor cells of 60 CRC patients and evaluated the relationship between DVL1 expression and liver metastasis status using immunohistochemical (IHC) staining.

## 2. Results and Discussion

### 2.1. Results

#### 2.1.1. Identification of Candidate Genes by Microarray Analysis and Bioinformatics

We utilized ten CRC cancer tissue specimens compared with their normal adjacent tissues. All results from the experiments underwent standardized analysis and validation. We then used GeneSpring Biological data analysis software, where hierarchical clustering was performed in the experimental groups to obtain an initial assessment of gene expression of all genes on the chip. After further analysis and validation, 1301 genes displayed overexpression. The 1301 overexpressed genes were analyzed with DAVID (Database for Annotation, Visualization, and Integrated Discovery) [18], and results indicated that a total of 31 genes were up regulated (gene expression ratio of cancer tissue to paired normal tissue > 2) and associated with CRC carcinogenesis and prognosis. The oligonucleotide sequences of 31 target genes have been listed in Table 1.

#### 2.1.2. Correlation between Liver Metastasis and Clinicopathological Features

We used data from a sample consisting of 116 men (54.2%) and 98 women (45.8%). The average age was 65.6 years (range 27–78 years). The clinicopathologic characteristics of these 214 International Union Against Cancer (UICC) stage I–III CRC patients have been listed in Table 2; 110 patients were subsequently diagnosed with stage I–II, and 104 with stage III. Overall, 41 of 214 (19.2%) patients were identified to have liver metastasis during follow-up, while 173 of 214 patients (80.8%) were found to have no detectable liver metastasis. Table 2 shows that there was no statistical significance between liver metastasis and gender, age, tumor size, stage, vascular invasion or perineural invasion (all *p* > 0.05).

#### 2.1.3. Correlation between *DVL1* Expression with Clinicopathological Data

A Chi-square test was used to determine the association between *DVL1* mRNA expression and clinicopathological parameters (including gender, age, tumor size, tumor stage, vascular invasion, perineural invasion and liver metastasis) of 214 CRC patients. We observed that a larger tumor size ≥ 5 cm (*p* < 0.0001), an advanced cancer stage (*p* = 0.002), the presence of vascular invasion (*p* < 0.0001), the presence of perineural invasion (*p* < 0.0001), and the presence of liver metastasis (*p* < 0.0001; Table 3) showed significant correlation. CRC patients with larger tumor size (70/98, (71.4%)) had significant *DVL1* mRNA overexpression than patients with small tumor size (28/98, (28.6%)). Stage III patients (59/98, (60.2%)) had significant *DVL1* mRNA overexpression than stage I and II patients (39/98, (39.8%)). Patients with vascular invasion (61/98, (62.2%)) had *DVL1* mRNA overexpression than patients without vascular invasion (37/98, (37.8%)) with statistical significance. CRC patients with perineural invasion (52/98, (71.4%)) had significant *DVL1* mRNA overexpression than patients without perineural invasion (46/98, (46.9%)). During follow-up period, *DVL1* mRNA was significantly overexpressed in liver metastasis patients (32/41, (78.0%)) than CRC patients without liver metastasis (66/173, (38.2%)).

#### 2.1.4. Correlation between *DVL1* Expression and Liver Metastasis

By univariate analysis, patients with *DVL1* overexpression had a risk of liver metastasis of 5.746 times greater than that of those without *DVL1* overexpression (OR: 5.764; 95% CI: 2.588–12.837; *p* < 0.0001). In Table 4, multivariate Cox proportional hazards regression analysis was performed to assess the significance of multiple predictors of liver metastasis in the follow-up period. The presence of *DVL1* mRNA overexpression (*p* = 0.006; OR, 3.768; 95% CI: 1.469–9.665) was demonstrated to be an independent predictor of postoperative liver metastasis (Table 4).

#### 2.1.5. Localization and Expression of DVL1 in Human CRC Tissue by Immunohistochemical Stain (IHC) and the Correlation with Clinicopathological Data

**Figure 1:**
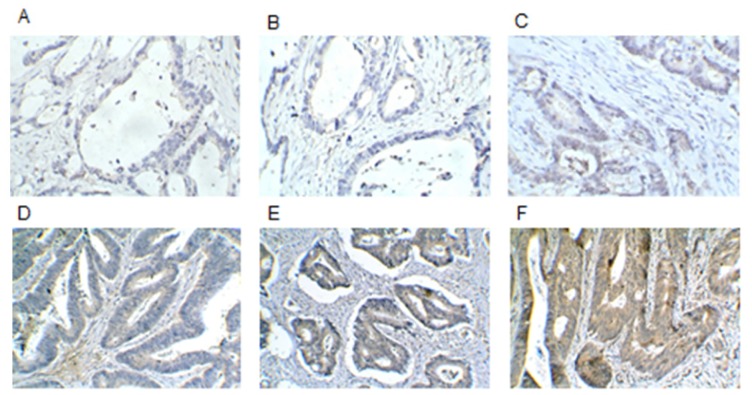
Immunohistochemistry (IHC) method with polyclonal mouse antibody against DVL1 was performed to detect DVL1 expression in 60 colorectal cancer (CRC) tumor specimens. (**A**) Blank; (**B**) Negative staining (score 0); (**C**) Weakly positive staining (score 2); (**D**) Weakly positive staining (score 6); (**E**) Strongly positive staining (score 8); (**F**) Strongly positive staining (score 12) in CRC tissues. Magnification, ×100.

We utilized DVL1 antibody to perform IHC staining in the collected tissue biopsies of 60 CRC patients, to localize and analyze DVL1 expression in CRC tissues. A positive yellowish-brown appearance after tissue dyeing indicated that DVL1 was overexpressed (Figure 1), and the immunoreactivity (yellowish-brown appearance) was localized in the cytoplasm of CRC tumor cells. From the correlation between DVL1 high expression and clinicopathologic features of 60 CRC patients using univariate analyses, we observed that a deeper tumor invasion (*p* = 0.036), the presence of perineural invasion (*p* = 0.028), and the presence of liver metastasis (*p* = 0.013) showed significant correlation (Table 5).

### 2.2. Discussion

In terms of incidence, CRC is the third-ranked cancer in males and the second-ranked in females [1], and the mortality rates are higher in metastasis cases [19]. As tumor invasion and metastasis ultimately affect cancer prognosis, it is important to predict the invasion potential of tumor cells. CRCs are characterized by aberrant gene expression signatures associated with disease initiation and progression. Therefore, identification of novel biomarkers in CRC invasion potential will be helpful in understanding and monitoring cancer progression and metastasis. By targeting these biomarkers, researchers can create new treatment modalities for the disease. For studies on prognosis-determining mechanisms using clinical samples, it is desirable to have patients that are subject to identical clinical conditions, including therapy, but this is very difficult to achieve in practice. In Taiwan, distant metastasis has an important meaning in relation to survival for CRC patients. We focus on liver metastasis-positive patients in order to avoid the influence of differing clinical conditions as much as possible in the present study.

In the past decade, the commonly used technique for the detection of nucleic acid material of CTCs was polymerase chain reaction, reverse-transcriptase PCR (RT-PCR), or real-time quantitative PCR (Q-PCR) assays, which permit sensitive detection of CTCs in peripheral blood. Due to the heterogeneity of gene marker expression in blood, a multi-marker assay is regarded as more reliable and sensitive than a single-marker assay [20,21]. Our previously well-established membrane array-based multi-marker assay can detect gene expression of CTCs in the peripheral blood of CRC patients; this was found to be a rational approach for the postoperative surveillance of CRC patients [22–25]. We demonstrated that this diagnostic technique was feasible and highly sensitive. A number as low as 5 cancer cells in 1 mL of blood could be distinctively detected, and the sensitivity, specificity and accuracy of the diagnostic membrane array were 83.7%, 90.9% and 86.8% [22]. In fact, we have demonstrated a significant correlation between real-time Q-PCR and the membrane array method in the detection of CTCs in CRC patients [10]. However, at the time of outcome reading, every gene was calculated by the same value which did not differentiate the influence of each gene for a specific disease prognosis, a major limitation of this technique in clinical applications [26]. In addition, the cost of the digoxigenin enzyme used on the colorimetric biochip platform was too high for laboratory diagnosis applications, and the high criteria of the operation techniques prevented its widespread availability for clinical applications. Therefore, we developed the next generation biochip operation platform (chemiluminescent membrane array), which replaced the conventional digoxigenin system with the biotin-avidin enzyme system to lower the cost [27]. The sensitivity, specificity, and accuracy were 90.2%, 94.9%, and 93.5%, respectively, and the detection limitation was three colon tumor cells per millimeter of blood [27]. Furthermore, we weighted multiples for each gene target in the membrane array to improve the CTC detection rate in order to establish a novel weighted enzymatic chip array (WEnCA) platform [13]. In current study, we applied WEnCA platform to investigate CTCs and candidate gene expression.

Because it was necessary to be able to select genes that actually did determine the prognosis, we selected the prognosis-determining function and its related functional pathway on the basis of database searches and published data. For our investigation, we selected 31 upregulated genes with differences in expression between Taiwanese CRC tumor specimens and paired normal adjacent tissues by cDNA microarray and these genes were close related to CRC carcinogenesis. A WEnCA including these 31 genes was used to investigate CTCs in 214 postoperative stage I–III CRC patients. Finally, this study demonstrated that *DVL1* gene overexpression was significantly associated with liver metastasis.

Previous studies have analyzed the relationship between a small number of genes and cancer malignancy and determined the importance of the Wnt signal pathway and apoptosis in cancer development [28]. The *DVL1* gene on chromosome 1p36 belongs to a family of highly conserved secreted proteins which regulates embryonic induction, generation of cell polarity and specification of cell fate through activation of Wnt signaling pathways [17]. Wnt signaling activates the gene encoding *DVL1*; the latter suppresses β-catenin by promoting its degradation through enhanced inactivation of glycogen-synthase-kinase 3. Increased expression of *DVL1* has been reported in primary breast cancers [29] and cervical squamous cell carcinomas [17]. *DVL1* was also reported to be overexpressed in breast cancer patients with a shorter distant metastasis free survival and a shorter overall survival [30]. However, there has been no system proven capable of accurately predicting outcomes as a clinical test.

Based on our research, we have not yet come across an investigation of *DVL1* expression pattern and its clinical significance in human colorectal adenocarcinoma. In our study, we demonstrate overexpression of *DVL1* mRNA in over 45% of primary CRCs (98 of 214 cases). In addition, IHC staining helped us identify the high immunoreactivity expression of DVL1 in the cytoplasm of tumor cells of 78.9% (15/19) CRC patients with liver metastasis. These data indicate that the amplification and increased expression of the *DVL1* gene may play some role in the development of metastasis through derangement of the Wnt signaling pathway.

## 3. Experimental Section

### 3.1. Clinical Samples Collection

For the microarray, fresh CRC tissue and paired adjacent non-tumor tissue samples were obtained from ten pathology-proven UICC stage I, II, and stage III CRC patients who underwent surgical resection at the Kaohsiung University Hospital between 2011 and 2012. These 10 patients included 6 males and 4 females (mean age, 57.8 ± 11.85 years). Patients with other malignant diseases in their medical history were excluded. After surgical resection, the fresh tissue samples were immediately immersed in RNAlater (Ambion Inc., Austin, Texas, USA) and stored overnight, at a temperature of 4 degrees to allow thorough penetration of the tissues; the samples were then frozen at minus 80 degrees until RNA extraction. Both, the tumor tissue and the adjacent non-tumor tissue, which was located more than 2 cm away from the CRC, were sampled and then verified by pathological examination.

Included in the peripheral blood CTC study were stage I–III CRC patients admitted to the Department of Surgery of Kaohsiung Medical University Hospital for elective surgery between January 2002 and December 2005. Patients with other malignant diseases in their medical history were excluded. Of the 221 stage I–III CRC patients, 4 who had surgical mortality, and 3 who reported a positive surgical resection margin for tumor invasion were excluded. The remaining 214 stage I–III CRC patients (116 males and 98 females, median age 59.5 ± 11.6 years) with curative resection for the primary lesion were enrolled for our study. Curative resection was defined as any gross residual tumor that did not remain in the surgical bed and in which the surgical resection margin was pathologically negative for tumor invasion. CTCs in peripheral blood of these 214 patients were detected using our WEnCA method. Postoperative surveillance consisted of medical history and physical examination every three months. Abdominal ultrasonography or computed tomography was performed every six months, and chest radiography and total colonoscopy were performed once a year. We followed up with patients at 3-monthly intervals for 2 years and 6-monthly intervals thereafter; median follow-up was 44 months (range 21–66 months). The development of new recurrent or metastatic lesions after operation was defined as a postoperative relapse. The type of postoperative relapse was designated as local recurrence (tumor growth restricted to the anastomosis or the region of primary operation) or distant metastases (distant metastases or diffuse peritoneal seeding). A 4-mL sample of peripheral blood was obtained from each CRC patient postoperatively (1 week after operation) for total RNA isolation. To prevent contamination of epithelial cells, peripheral blood samples were obtained through a catheter inserted into a peripheral vessel, and the first 5 mL of blood was discarded.

For IHC study, archival formalin-fixed, paraffin embedded (FFPE) tissue specimens from 60 primary CRC patients who underwent surgical resection at Kaohsiung Medical University Hospital from December 2002 to December 2006 was recruited. We included patients who met the following eligibility criteria: (1) diagnosis of colorectal adenocarcinoma identified by histopathological examination; (2) surgical history that included radical tumor resection plus lymphadenectomy (limited or extended); (3) availability of complete follow-up data; (4) no preoperative treatment, such as chemotherapy and radiotherapy; and (5) no history of familial malignancy or other synchronous malignancy (such as esophageal cancer, and gastric cancer). Tumor resection and lymphadenectomy were performed by experienced surgeons and we found similar results in all patients who underwent radical resections. These patients included 38 males and 22 females, with a median age of 55 years (range, 26–75 years). Each tumor sample was assigned a histological grade based on the World Health Organization (WHO) classification criteria.

All patients were staged using the 7th edition of the International Union Against Cancer (UICC) Tumor-Node-Metastasis (TNM) staging system. Written informed consent was obtained from each subject and/or guardian. Sample acquisition and subsequent use were also approved by the hospital’s institutional review board.

### 3.2. Total RNA Extraction and First Strand cDNA Synthesis

Total RNA was extracted from the fresh whole blood of postoperative CRC patients using the GeneCling^®^ Enzymatic Gene Chip Detection Kit (MedicoGene Biotechnology Co., Ltd., Los Angeles, CA, USA). RNA purified is quantified by OD 260 nm using an ND-1000 spectrophotometer (NanoDrop Technologies, Wilmington, DE, USA) and quantitated by Bioanalyzer 2100 (Agilent Technologies, Santa Clara, CA, USA). First-strand cDNA was synthesized from total RNA, using a GeneCling^®^ Enzymatic Gene Chip Detection Kit. The reverse transcription was carried out in a reaction mixture consisting of a 3 μg/mL oligo (dT) 18-mer primer, 1 μg/mL random 6-mer primer, 100 mmol/L deoxyribonucleotide triphosphate, 200 units of Reverse Transcriptase MMLV, and 25 units of ribonuclease inhibitor. The reaction mixtures with RNA were incubated at 42 °C for 2 h minimum, heated to 95 °C for 5 min, and then stored at −80 °C until analysis.

### 3.3. Oligonucleotide Microarray Analysis

The oligonucleotide array contains 22,500 elements designed for expression profiling (Human 1A V2, Agilent Technologies, Palo Alto, CA, USA), for which over 18,000 well-characterized, full-length human genes have been defined. First-strand cDNA targets for hybridization were made by reverse transcription of the mRNA isolated from ten CRC cancer tissue specimens and their normal adjacent tissues by using SuperScript II RT (Gibco-BRL, Gaithersburg, MD, USA) in the presence of either Cy3- or Cy5-labeled dUTP (Amersham Pharmacia Biotech, Piscataway, NJ, USA). The targets were dried to 18 μL by a SpeedVac™ concentrator (Thermo Electron Co, Waltham, MA, USA), and 3.6 μL 20 × SSC, 1.8 μL 10 mg/mL poly-A and 0.54 μL 10% SDS were added. The mixture was then heated upto 100 °C for 2 min proceeding to the hybridization reaction on Human 1A Oligo Microarray V2 array slides (Agilent Technologies, Santa Clara, CA, USA) in an incubator at 60 °C for 17 h. After being sequentially washed with 1 × SSC, 0.2 × SSC and 0.5 × SSC, hybridized microarray slides were scanned and fluorescence signals were detected by using an Axon GenePix Pro 3.0™ (Axon Instruments, Foster City, CA, USA). The acceptance criterion for a gene signal was a signal-to-noise ratio of ≥ 2. If either the Cy3 or Cy5 signal of a specific spot passed the criterion, the flag of its ratio was counted to be “True”. The element with the “True” flag was analyzed with GeneSpring GX7 (Silicon Genetics, Redwood City, CA, USA). The differentially expressed elements were analyzed by the two-sided statistical tolerance interval (95%).

### 3.4. Preparation of Biotin-Labeled cDNA Targets and Hybridization

**Figure 2:**
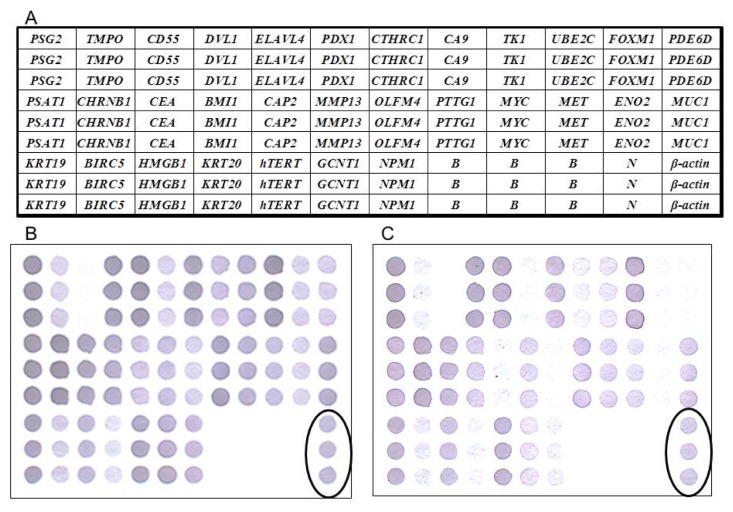
(**A**) The schematic representation of a weighted enzymatic chip array (WEnCA) with 31 candidate genes, one positive control (*β-actin*), one negative control (*Oryza sativa* sequence), and the blank control (dd water). Oligonucleotide fragments are blotted on membranes in triplicate. The expression levels of each gene spot were quantified then normalized based on reference gene (*β-actin*) density. We defined an overexpressed gene spot as occurring when the normalized spot density was 2 or more. Results of WEnCA of CRC with liver metastasis (**B**) and without liver metastasis (**C**). Circle: positive control.

First-strand cDNA targets for hybridization were made by reverse transcription of the mRNA from the tumor and corresponding normal tissues of LARC patients in the presence of Biotin-labeled UTP by using a GeneCling^®^ Enzymatic Gene Chip Detection Kit. The hybridized arrays were then scanned with an Epson Perfection 1670 flatbed scanner (SEIKO EPSON Corp., Nagano-ken, Japan). Subsequent quantification analysis of the intensity of each spot was carried out using AlphaEase^®^ FC software (Alpha Innotech Corp., San Leandro, CA, USA). Spots consistently carrying a factor of two or more were considered to be differentially expressed. A deformable template extracted the gene spots and quantified their expression levels by determining the integrated intensity of each spot after background subtraction. The fold ratio for each gene was calculated as follows: spot intensity ratio = mean intensity of target gene/mean intensity of *β-actin*. Figure 2 provides the schematic representation of the membrane array with 31 candidate genes, one positive control (*β-actin*), one negative control (*Oryza sativa* sequence), and the blank control (dd water).

### 3.5. Weighted Enzymatic Chip Array (WEnCA)

The procedure of the membrane-array method for gene detection was performed based on our previous study [22]. Visual OMP3 (Oligonucleotide Modeling Platform, DNA Software, Ann Arbor, MI, USA) was used to design probes for target genes and β-actin (Table 1). The newly synthesized oligonucleotide fragments were dissolved in distilled water to a concentration of 100 mM, and applied to a BioJet Plus 3000 nL dispensing system (BioDot Inc., Irvine, CA, USA), which blotted the target oligonucleotide; the β-actin control was used sequentially (0.05 μL per spot and 1.5 mm between spots) on a SuPerCharge nylon membrane (Schleicher and Schuell, Dassel, Germany) in triplicate. After rapid drying and cross-linking procedures, the preparation of the membrane array was accomplished. The expression levels of each gene spot measured by the WEnCA method were quantified and then normalized based on reference gene (β-actin) density. We have defined as an overexpressed gene spot as a case where the observed normalized spot density was 2 or more.

### 3.6. Immunohistochemistry

Eighteen CRC tissue specimens with liver metastasis and 42 specimens without liver metastasis were stained using standard IHC methods. We used a polyclonal mouse antibody against DVL1 (human homolog of the Drosophila dishevelled gene; dilution 1:100, Abnova, Taipei, Taiwan). Microwave antigen retrieval was performed in citrate buffer (pH 6.0). For antigen visualization, we used the EnVision/HRP system and DAB+ (Dako, Glostrup, Denmark). Slides were subsequently counterstained with hematoxylin, dehydrated and mounted in Canadian balsam. The IHC procedure was optimized by testing different antigen retrieval methods and using negative controls. We regarded cells as immunoreactive when an obvious cytoplasmic (DVL1) staining was seen. Immunoreactivity was scored as follows: retained (+) when more than 50% of cytoplasm was strongly positive, and absent (−) when 0%–50% of the cytoplasm was positive. Immunoreactivity was evaluated according to the following standards: staining intensity was classified as 0 (lack of staining), 1 (weak color), 2 (distinct color) or 3 (dense, dark, strong color), and the percentage of staining was designated 1 (less than 20%), 2 (20%–50%), 3 (51%–80%) or 4 (>80%). For each section, the IHC score was calculated by multiplying these two values (which ranged from score 0 to score 12) and the result was defined as low expression (score 0 to score 6) and high expression (score 7 to score 12).

### 3.7. Statistical and Database Analysis

All data were statistically analyzed using the Statistical Package for the Social Sciences, version 12.0 (SPSS Inc., Chicago, IL, USA). A *p*-value less than 0.05 was considered statistically significant. A Chi-square test was used to determine the association between *DVL1* expression and clinicopathological parameters. Multivariate Cox proportional hazards regression analysis was performed to assess the significance of multiple predictors of liver metastasis. The association between clinicopathological features and DVL1 IHC status were compared using Fisher’s exact test because the expected values are small than 5.

## 4. Conclusions

In conclusion, we found that liver metastasis was significantly correlated with overexpression of *DVL1*. The *DVL1* overexpression may affect metastatic behavior of tumor cells in CRC patients. *DVL1* gene may preoperatively be a suitable new marker for CRC prognosis and liver metastasis. This biomarker can predict disease prognosis, and also aid in the making of appropriate strategic treatment decisions in CRC.

## Figures and Tables

**Table 1:** Oligonucleotide sequences of 31 target genes.

Gene	Oligonucleotide sequences (5′ → 3′)
*PSG2*	CTCGGAAACTTTTGGTGGCTGGGCTTCAATCGTGACTTGGGCAGT
*TMPO*	TGCAGCCCCTTTGATTGGTCTGCGGCAACTAGCACTAATTCCTGT
*CD55*	AGTTGCAGTAAGCAGAAGCCTCGTGGCTCCGCAATACCAGTTAAA
*DVL1*	GAGCGTGACAGTGACGATGTTGAGGGACATGGTGGAGTCGGTTAT
*ELAVL4*	TTGCCCCTGTTTGCATGGGAGAAGGACAGTTTCTGTTGTTGCTGG
*PDX1*	TAGAAAGTTCCGAATATCTTTGGTTTAGTGGTCACCCAGGGGTCC
*CTHRC1*	CAACCCAGATAGCAACATCCACTAATCCAGCACCAATTCCTTCAC
*CA9*	CCTTCCTCAGCGATTTCTTCCAAGCGAGACAGCAACTGCTCATAG
*TK1*	CAGGGAGAACAGAAACTCAGCAGTGAAAGCCGCAGAGGGGAAGAA
*UBE2C*	AACTTCACTGTGGGCGCATTGTAAGGGTAGCCACTGGGGAACTCT
*FOXM1*	ACTTGGGGCATTTTGAACAGGAAGGGGCAGCCTCCGTCTTTTGAG
*PDE6D*	TGCCAGAGTATCTTCCCTGTCTCAGCATCCCGAAGGTTCATCCAA
*PSAT1*	TTGACCTTGAATCAACAGCCGCTGAACCCAGGAGACCCCACAGAT
*CHRNB1*	TAGGGTCCCAACGCTGGTGAAGATGATGAAAGTCCACAGGAAGAG
*CEA*	TGGTGGGCGGGTTCCAGAAGTTTAGAAGTGAGGCTGTGAGCAGAA
*BMI1*	CGAGGTCTATTGGCAAAAGAAGATTGGTGGTTACCGCTGGGGCTG
*CAP2*	ACATGGCGGAGCCCTTTTGTAATTGCTTCTCCCTGGTTAAGTTGG
*MMP13*	AAAGTGGCTTTTGCCGGTGTAGGTGTAGATAGGAAACATGAGTGC
*OLFM4*	AGCAGGTGCCTCATCTACAGATCCTTCTGGGATTTATTTGCCATG
*PTTG1*	TATCTATGTCACAGCAAACAGGTGGCAATTCAACATCCAGGGTCG
*MYC*	CTGACCTTTTGCCAGGAGCCTGCCTCTTTTCCACAGAAACAACAT
*MET*	CCCGAGTTCTTTCTATTGATGCGTTCATGCTCTTGACCCTGGTAG
*ENO2*	CCTTTCTATGACCCTTCCCATTCTAGCAAGACCTCCCACCCCAGT
*MUC1*	TCTTTCGGCGGCACTGACAGACAGCCAAGGCAATGAGATAGACAA
*KRT19*	ACCTTGGAGGCAGACAAATTGTTGTAGTGATCTTCCTGTCCCTCG
*BIRC5*	CTCTAACCTGCCATTGGAACCTCACCCATAGCCCAGAAGCCTCAT
*HMGB1*	ATTGCAGCCTATCACTAACCCTGCTGTTCGCTTGCATGTATCTTG
*KRT20*	GGGCGTTGGTTTCGTACCACTGCTTGATTTGCACTTCAAGTTTGG
*hTERT*	AGGGGTGAACAATGGCGAATCTGGGGATGGACTATTCCTATGTGG
*GCNT1*	GTGTTTGTCAGCTTTCCATTAACGACCTCATACCGCTTCTTCCAC
*NPM1*	TTTGTCTCCCCACCATTTCCATGTCTGACCACCGCTACTACTATG
*Oryza sativa*	CTCGGTAACCTCTCATTCCTCTACACCCTCGACCTCACCAACACCAGCCT
*β-actin*	ATGCTCGCTCCAACCGACTGCTGTCACCTTCACCGTTCCAGTTTT

**Table 2:** Correlations between clinicopathological features and liver metastatic status for 214 postoperative colorectal cancer patients.

Characteristics	Total cases	Liver metastasis N (%)	No Liver metastasis N (%)	*p*-Value *
Gender				
Male	116	20 (17.2)	96 (82.8)	0.488
Female	98	21 (21.4)	77 (78.6)	
Age (years)				
<60	73	15 (20.8)	58 (80.6)	0.714
≥60	141	26 (18.3)	115 (81.0)	
Tumor size a				
<5 cm	109	20 (18.3)	89 (81.7)	0.862
≥5 cm	105	21 (20.0)	84 (80.0)	
Stage (UICC) b				
I–II	110	19 (17.3)	91 (82.7)	0.492
III	104	22 (21.2)	82 (78.8)	
Vascular invasion				
Positive	90	18 (20.0)	72 (80.0)	0.861
Negative	124	23 (18.5)	101 (81.5)	
Perineural invasion				
Positive	85	11 (12.9)	74 (87.1)	0.076
Negative	129	30 (23.3)	99 (76.7)	

a Tumor size was measured for invasive area by histological examination;

b UICC: The American Joint Commission on Cancer/International Union Against Cancer (AJCC/UICC, 2002);

* *p*-value is tested by chi-square tests; *p*-value < 0.05 is significant.

**Table 3:** The relationship between clinicopathological features and *DVL1* mRNA overexpression in 214 postoperative colorectal cancer patients.

Characteristics	Total case	*DVL1*		*p*-Value *

		+ (*n =* 98) (%)	− (*n =* 116) (%)	
Gender				
Male	116	55 (56.1)	61 (52.6)	0.605
Female	98	43 (43.9)	55 (47.4)	
Age (y/o)				
<60	73	34 (34.7)	39 (33.6)	0.869
≥60	141	64 (65.3)	77 (66.4)	
Tumor Size				
<5 cm	109	28 (28.6)	81 (69.8)	<0.0001
≥5 cm	105	70 (71.4)	35 (30.2)	
Stage (UICC) a				
I–II	110	39 (39.8)	71 (61.2)	0.002
III	104	59 (60.2)	45 (38.8)	
Vascular invasion				
Positive	90	61 (62.2)	29 (25.0)	<0.0001
Negative	124	37 (37.8)	87 (75.0)	
Perineural invasion				
Positive	85	52 (53.1)	33 (28.4)	<0.0001
Negative	129	46 (46.9)	83 (71.6)	
Liver metastasis				
Yes	41	32 (32.7)	9 (7.8)	<0.0001
No	173	66 (67.3)	107 (92.2)	

a UICC: The American Joint Commission on Cancer/International Union Against Cancer (AJCC/UICC, 2002);

* *p*-value was tested by chi-square tests; *p*-value < 0.05 is significant.

**Table 4:** Multivariate analysis of clinicopathological factors, *DVL1* mRNA expression and liver metastasis status for 214 postoperative colorectal cancer patients.

Parameters	Liver metastasis (*n =* 41) (%)	No liver metastasis (*n =* 173) (%)	Multivariate analysis Odd ratio (95% CI)	*p*-Value *
Sex (Male/Female)	20 (48.8)/21 (51.2)	96 (55.5)/77 (44.5)	0.726 (0.289~1.828)	0.497
Age (≥60/<60)	26 (63.4)/15 (38.6)	115 (66.5)/58 (33.5)	1.241 (0.483~3.186)	0.654
Tumor size (≥5 cm/<5 cm)	21 (51.2)/20 (48.8)	84 (48.6)/89 (51.4)	1.076 (0.416~2.786)	0.880
Stage (UICC) a (III/I–II)	22 (53.7)/19 (46.3)	82 (47.4)/91 (52.6)	0.859 (0.201~3.663)	0.837
Vascular invasion (yes/no)	18 (43.9)/23 (56.1)	72 (41.6)/101 (58.4)	0.168 (0.030~0.929)	0.641
Perineural invasion (yes/no)	11 (26.8)/30 (73.2)	74 (42.8)/99 (57.2)	1.499 (0.439~5.117)	0.518
*DVL1* overexpression (yes/no)	32 (78.0)/9 (22.0)	66 (38.2)/107 (61.8)	3.768 (1.469~9.665)	0.006

a UICC: The American Joint Commission on Cancer/International Union Against Cancer (AJCC/UICC, 2002);

* *p*-value was tested by multivariate Cox proportional hazards regression analysis and the *p*-value was adjusting for age, sex, tumor size, stage, vascular invasion, and perineural invasion.

**Table 5:** Immunohistochemical staining of DVL1 in 60 CRC patients’ tumor specimens.

Characteristics	DVL1 IHC		*p*-Value

	High (*n =* 33)	Low (*n =* 27)	
Gender			
Male	22	16	0.554
Female	11	11	
Age (y/o)			
<60	11	6	0.342
≥60	22	21	
Tumor Location			
Colon	26	25	0.166
Rectum	7	2	
Tumor Size			
<5 cm	22	12	0.084
≥5 cm	11	15	
Depth			
T1 + T2	0	4	0.036 *
T3 + T4	33	23	
Stage (UICC) a			
III	32	26	1.000
IV	1	2	
N stage b			
N1	19	19	0.306
N2	14	8	
Vascular invasion			
Positive	11	10	0.765
Negative	22	17	
Perineural invasion			
Positive	15	5	0.028 *
Negative	18	22	
Liver metastasis			
Yes	15	4	0.013 *
No	18	23	

a UICC: The American Joint Commission on Cancer/International Union Against Cancer (AJCC/UICC, 2002);

b N1: Metastasis to 1 to 3 regional lymph nodes, N2: Metastasis to 4 or more regional lymph nodes;

* *p*-value < 0.05 is significant.
